# Nocebo response intensity and influencing factors in the randomized clinical trials of irritable bowel syndrome: A systematic review and meta-analysis

**DOI:** 10.3389/fmed.2022.1018713

**Published:** 2022-12-20

**Authors:** Ruijie Li, Fuping Chen, Xuanxuan He, Yuqing Feng, Qiaoqiao Pei, Dongke Wang, Xinghuang Liu, Jinsong Liu, Xiaohua Hou, Tao Bai

**Affiliations:** Division of Gastroenterology, Union Hospital, Tongji Medical College, Huazhong University of Science and Technology, Wuhan, Hubei, China

**Keywords:** nocebo response, irritable bowel syndrome, adverse event (AE), systematic review, meta-analysis

## Abstract

**Objective:**

To estimate the magnitude of the nocebo response and explore its influencing factors in irritable bowel syndrome (IBS).

**Methods:**

The PubMed, Embase, and Cochrane Library databases were searched up to March 2021. We performed a random effects meta-analysis of the proportion of adverse events (AEs) in placebo-treated patients with IBS who are involved in parallel-designed, randomized, placebo-controlled trials investigating pharmacological interventions and evaluated the effect of trial characteristics on the magnitude of the nocebo response rate.

**Results:**

A total of 6,107 studies were identified from the databases. After evaluation, 53 met the eligibility criteria and were included. The overall pooled nocebo response rate was 32% (95% CI: 26–38%). The most commonly reported AEs were headache (9%), nasopharyngitis (7%), abdominal pain (4%), and nausea (4%). The nocebo response rate was low compared with that in the treatment group applying probiotics, antispasmodics, and Traditional Chinese medicine, but high compared with that in antibiotic treatment group. The nocebo rate in patients using diaries to record AEs was lower than the average, and was higher in patients recording through checkup.

**Discussion:**

Patients with IBS have significant nocebo response intensity in clinical trials. Based on findings in this study, we recommend the researchers pay attention to the common AEs and carefully analyze the relation to the intervention.

## Introduction

Irritable bowel syndrome (IBS) is a chronic, functional gastrointestinal disorder characterized by a symptom complex of abdominal pain or discomfort and altered bowel habits that present as diarrhea or constipation yet without abnormal morphological, histological, or inflammatory markers (1). As a common recurrent functional gastrointestinal disorder, it seriously affects the physical and mental health of patients. In addition to the discomfort and painful experience, IBS also has a great negative impact on the quality of life. Compared with non-IBS patients, patients with IBS are more likely to be absent from work or school, and have varying degrees of reduced productivity and activity at work (2). Some patients may also have different degrees of mental disorders, such as anxiety, depression, tension, etc. (3). At the same time, the cost of living for IBS patients is significantly higher than that for non-IBS patients (4). In recent years, as the incidence of IBS has been on the rise worldwide, the burden on the whole society has become increasingly prominent (5, 6). Therefore, the research and development of more effective clinical drugs is imminent, and the clinical efficacy of both commonly used traditional drugs and newly developed drugs still needs to be further explored.

As is known to all, the establishment of placebo control in clinical trials of new drugs requires informed consent of patients. In recent years, with the gradual deepening of the study on placebo effect, more and more people have noticed that informed consent also plays an important role in the occurrence of adverse reactions. Under the informed consent, doctors would explain possible benefits and risks of tested drugs and specific placebo-controlled methods before the trials. However, the disclosure of these related experimental designs itself may induce adverse effects through an anticipatory mechanism, known as the nocebo effect (7).

Noticeable nocebo effect may lead to the inaccurate estimates of adverse events (AEs) associated with treatment, which may occur through increasing the proportion of AEs in placebo group or increasing the ratio of AEs that are unrelated to the intervention among patients in the intervention group (8). Moreover, nocebo effect has a negative impact on patients’ treatment compliance, meta-analyses of randomized controlled trials (RCTs) of patients with rheumatic and musculoskeletal diseases (RMDs) indicate that withdrawal of treatment by placebo-arm participants due to AEs is common, which suggests that nocebo effect can have a notable influence on patients’ medication adherence (9). Nocebo response also reduces patients’ confidence in the efficacy of follow-up treatment and endangers the durability of treatment, thus adversely affecting the trial effect (10), and causing bias in the efficacy evaluation of the whole clinical drug. Therefore, the nocebo effect has indispensable implications for drug development and RCT study design.

It is generally believed that minimizing the nocebo effect contributes to better treatment outcomes and fewer side effects (11). Since nocebo effect is related with accumulated experience of past diseases, especially when patients require multiple treatments to control the disease (8), it may interfere with drug therapy trials for functional gastrointestinal diseases more seriously. Therefore, in the clinical trials for IBS, we should actively explore the method to minimize the nocebo effect, and to make the best effort to specify the intensity of nocebo effect of currently tested drugs in order to get more accurate curative effect observation and more authentic safety assessment of clinical medicine, avoiding manpower and material resources waste as well as financial loss.

The purpose of this study is to evaluate the intensity and influencing factors of nocebo effect in randomized clinical trials for IBS through systematic review and meta-analysis, which could guide investigators to better conduct clinical practice and design research trials for drug safety evaluation.

## Methods

### Eligibility criteria

We included placebo-controlled, parallel-designed RCTs that investigated any pharmacological intervention in adult patients (age ≥ 18 years) with IBS. We considered an RCT eligible only when it compared a pharmacological agent with a placebo arm and assessed AEs in both groups. We excluded studies that reported only AE rates and not the specific number of AE, studies in which the participants were taking drugs that affect digestive function, studies with repeated reports, and studies with design flaws.

### Data sources and searches

We searched the PubMed, Embase, and Cochrane Library to identify potential RCTs from inception to March 2021.

We used “random controlled trial,” “placebo,” “sham,” “dummy” in combination with “irritable bowel syndrome” and “drug therapy” as the keywords for our search strategy. The detailed search strategy is provided in Supplementary Digital Content 1 (see Supplementary Figure 1).

### Study selection

Record retrieval was conducted independently by two reviewers (RL and FC) according to the Cochrane Handbook. These two reviewers independently screened both titles and abstracts for eligibility based on the inclusion and exclusion criteria described above. Then, full-text screening was performed to finalize the included records. Records management was performed using Noteexpress 3.5.0.

### Data extraction

The full text of each eligible article was reviewed, and the related data were extracted by two reviewers (RL and Xu H) independently. We extracted demographic characteristics, including the name of the author, year of publication, sample size of the placebo arm, mean patient age, sex, duration of intervention, trial location, Numbers of centers, Rome diagnostic criteria, sponsorship, diagnostic types, dosing schedule and types of drugs from the articles. For the safety assessment, we extracted the event rate as the number of patients experiencing a certain AE in the placebo arm of all trials. We extracted common AEs, including constipation, diarrhea, vomiting, abdominal pain, dizziness, headache, skin rash, and upper respiratory tract infections. To explore the potential sources of heterogeneity and possible factors affecting the overall results, we stratified and performed subgroup analyses of 36 studies according to the Guidelines for Interpreting Subgroup Analysis.

### Risk of bias

We used the Cochrane risk-of-bias tool to classify studies as being at low, high, or unclear risk of bias in the following domains: randomization, allocation concealment, blinding of participants and personnel, blinding of the outcome assessment, incomplete outcome data, and selective reporting. Two researchers (FC and XuH) independently assessed the risk of bias, and disagreements were resolved by a third reviewer (TB). We rated studies as having a high or low overall risk of bias using previously defined criteria.

### Data synthesis and statistical methods

R 4.0.3^1^ was used to calculate the pooled nocebo response rate as well as its 95% confidence interval (95% CI), draw a forest plot, and perform all the statistical analyses. We performed a systematic review and meta-analysis of different AEs. The heterogeneity among all the included studies was assessed using *I*^2^. Due to the high heterogeneity of the included studies, we selected the random effects model.

## Results

### Basic characteristics of eligible studies and risk of bias

**FIGURE 1:**
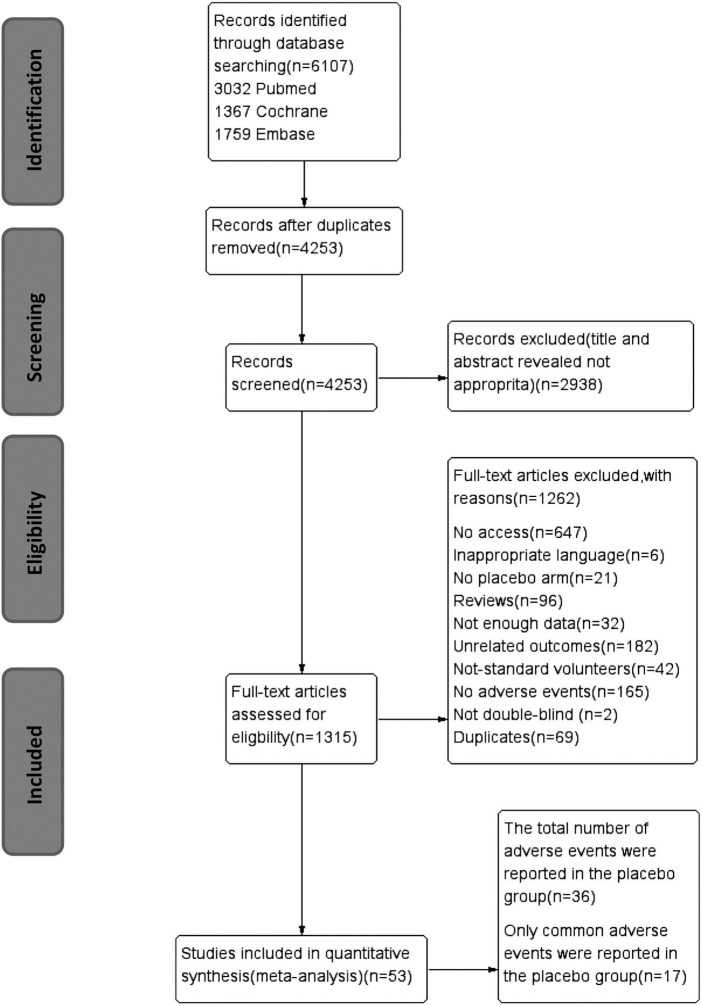
PRISMA flow diagram of trial inclusion. PRISMA, preferred reporting items for systematic reviews and meta-analyses; RCT, randomized controlled trial.

After screening and eligibility assessment, 53 studies were ultimately included (Figure 1). Overall AEs were reported in 36 studies, and 17 other studies reported simply individual AEs. In these 53 studies, 6,462 participants were allocated to placebo arms. The average age of the patients in placebo arms was 41.1 years and 54.9% of them were from North America. In total, 77.8% of studies were funded and 66.8% were carried out in multiple centers. In total, 61.1% of the trials had a duration of over 8 weeks and 41.7% of trials give patients drugs once a day (Table 1).

**TABLE 1:** Characteristics of 36 trials.

No.	References	Mean age (SD)/(range)	Sample size of the placebo arm	Male no. in the placebo arm	Duration of therapy (week)	Sponsorship	Trial location	No. of centers	Dosing schedule	Types of medication	Type of diagnosis	Rome	Extraction
1	Schutze et al. (27)	45 (18–73)	48	8	12	No	Austria	Multicenter	t.i.d.	Cisapride	IBS	Rome I	Diary
2	Bardhan et al. (28)	43 ± 14	117	33	12	No	UK	Multicenter	b.i.d.	Alosetron	IBS	Rome II	Follow-up
3	Novick et al. (29)	41 (11.7)	752	0	12	No	America	Multicenter	b.i.d.	Tegaserod	IBS	Rome I	Multiple ways
4	Kellow et al. (30)	36 (12.4)	261	29	12	Yes	The Asia-Pacific region	Multicenter	b.i.d.	Tegaserod	IBS	Rome II	Diary
5	Lu et al. (31)	41.2	17	0	16	No	Singapore	Single center	q.d.	Melatonin	IBS	Rome II	Diary
6	Vahedi et al. (32)	32.8 ± 9.5	22	11	12	Yes	Tehran	Single center	q.d.	Fluoxetine	IBS-C	Rome II	Follow-up
7	Saha et al. (33)	22 (19–68)	9	6	8	No	India	Single center	q.d.	Melatonin	IBS	Rome II	Unknown
8	Houghton et al. (34) A	(21–41)	12	4	3	Yes	UK	Single center	t.i.d.	Oral pregabalin	IBS	Rome II	Follow-up
9	Sabate et al. (35)	41.0 (11.1) (29–56)	15	0	10	No	France	Single center	b.i.d.	Tegaserod	IBS-C	Rome II	Unknown
10	Silk et al. (36)	54	14	0	12 weeks	Yes	UK	Single center	q.d.	*Trans-*galactooligosacc -haride prebiotic	IBS-D IBS-C IBS-A	Rome II	Multiple ways
11	Lembo et al. (37)	43.6 (18–65)	600	0	12 weeks	Yes	US	Multicenter	b.i.d.	Renzapride	IBS-C	Rome II	Checkup
12	Guglielmetti (38)	40.98 ± 12.80	62	21	4 weeks	Yes	Germany	Multicenter	q.d.	*Bifidobacterium bifidum* MIMBb75	IBS-D IBS-C IBS-A	Rome III	Unknown
13	Zakko (39)^†^	48.1 (12.8)	50	0	2 weeks	Yes	US Canada	Multicenter	b.i.d.	DNK333	IBS-D	Rome II	Unknown
14	Zakko et al.(39)^†^	44.5 (12.3)	78	0	4 weeks	Yes	US Canada	Multicenter	b.i.d.	DNK333	IBS-D	Rome II	Unknown
15	Tack et al. (40)	45.7 (14.6)	59	22	8 weeks	Yes	US Belgium	Multicenter	t.i.d.	AST-120	Non-constipating IBS-D	Rome III	Unknown
16	Dove et al. (41)	44.6 (12.5)	159	49	12 weeks	No	US	Single center	b.i.d.	Eluxadoline	IBS-D	Rome III	Diary
17	Fukudo et al. (42)	40.2 ± 10.1	149	149	12 weeks	Yes	Japan	Multicenter	q.d.	Ramosetron	IBS-D	Rome III	Follow-up
18	Chmielewska-Wilkon et al. (43)	47.8 ± 13.1	23	7	4 weeks	No	Poland	Multicenter	t.i.d.	Otilonium bromide	IBS	Rome II	Diary
19	Sisson (44)	36.8 (10.8)	62	17	12 weeks	Yes	UK	Single center	q.d.	Symprove	IBS-D IBS-C IBS-A	Rome III	Unknown
20	Schoenfeld et al. (45)	46.2 (14.5) (18–82)	829	239	6	Yes	US Canada	Multicenter	t.i.d.	Rifaximin	IBS-D	Rome II	Checkup
21	Nielsen et al. (46)	50.4 (23.6–67.5)	25	16	14	Yes	Sweden	Single center	q.d.	PPC-5650	IBS	Rome III	Unknown
22	Cash et al. (47)	40.7	37	11	4	Yes	USA	Single center	t.i.d.	Peppermint Oil	IBS-M or IBS-D	Rome III	Diary
23	Mosaffa-Jahromi et al. (48)	32.35 ± 7.24	40	22	4	Yes	Iran	Single center	t.i.d.	Anisencap, colpermin	IBS	Rome III	Diary
24	Fukudo et al. (49)	41.5 ± 12.0	284	0	12	Yes	Japan	Multicenter	q.d.	Ramosetron	IBS-D	Rome III	Diary
25	Spiller et al. (50)	45.4 ± 14.1	187	31	12	Yes	France	Multicenter	b.i.d.	*Saccharomyces cerevisiae* CNCM I-3856	IBS	Rome III	Follow-up
26	Lembo et al. (51)	45.6 ± 13.8	308	89	51	Yes	United States and Europe	Multicenter	t.i.d.	Rifaximin	IBS-D	Rome III	Unknown
27	Fant et al. (52)	46.1 (13.7)	981	334	52	yes	United States	Multicenter	b.i.d.	Eluxadoline	IBS-D	Rome III	Follow-up
28	Ida et al. (53)	40.9 ± 11.1	51	51	12	Yes	Japan	Multicenter	q.d.	Ramosetron	IBS-D	Rome III	Follow-up
29	Fukudo et al. (54)	42.8 ± 12.1	102	0	12	Yes	Japan	Multicenter	q.d.	Ramosetron	IBS-D	Rome III	Diary
30	Whitehead et al. (55)	47.8 ± 12.5	63	0	4	Yes	USA	Multicenter	q.d.	ONO-2952	IBS-D	Rome III	Unknown
31	Ida et al. (56)	40.9 ± 11.1	51	51	12	Yes	Japan	Multicenter	q.d.	Ramosetron	IBS-D	Rome III	Follow-up
32	Fan et al. (57)	36.6 (35.3–37.9)	348	144	4	Yes	China	Multicenter	t.i.d.	Tongxie formula	IBS	Rome III	Unknown
33	Fukudo et al. (58)	41.6 ± 10.8	112	12	12	Yes	Japan	Multicenter	q.d.	Linaclotide	IBS-C	Rome III	Unknown
34	Yang et al. (59)	41.3	422	67	12	Yes	China, North America, and Oceania	Multicenter	q.d.	Linaclotide	IBS-C	Rome III	Follow-up
35	Chen et al. (60)	32.7 ± 8.2	80	31	4	Yes	China	Single center	t.i.d.	TXYF granules	IBS-D	Rome III	Diary
36	Skrzydlo-Radomanska et al. (61)	36.7 ± 12.7	33	9	8	Yes	Finland	Multicenter	b.i.d.	Synbiotic preparation containing Lactobacillus and Bifidobacterium probiotic strains and short chain Fructooligo-saccharides	IBS-D	Rome III	Follow-up

^†^There were two trials in one paper.

**FIGURE 2:**
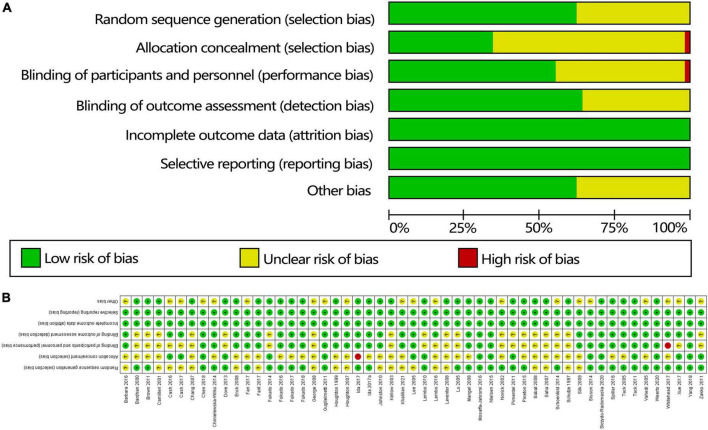
Quality of the studies based on the Cochrane risk-of-bias assessment tool: **(A)** risk-of-bias summary and **(B)** risk-of-bias table.

A risk-of-bias graph (Figure 2) and risk-of-bias summary were generated to evaluate seven risk-of-bias parameters for the whole study and for each study.

### Nocebo response rate

**FIGURE 3:**
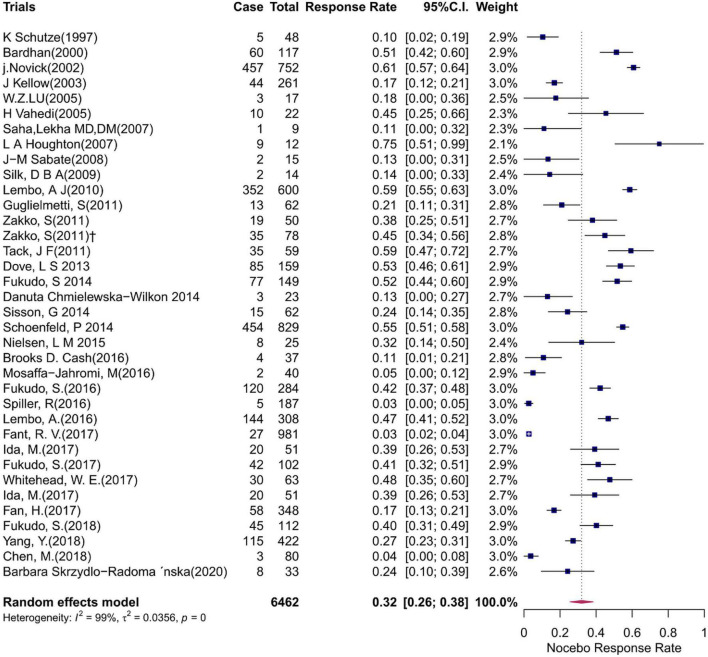
Forest plot for the pooled analysis.

The pooled nocebo response rate was 32% (95% CI: 26–38%), with significant heterogeneity among trials (*I*^2^ = 99%, *P* < 0.01). The individual nocebo response rate of each study varied from 3 to 75%. A forest plot for the pooled analysis is shown in Figure 3. Serious adverse events (SAEs) were reported in 0.74% of placebo-treated patients, and dropouts due to AEs were reported in 0.46% of the trials.

**FIGURE 4:**
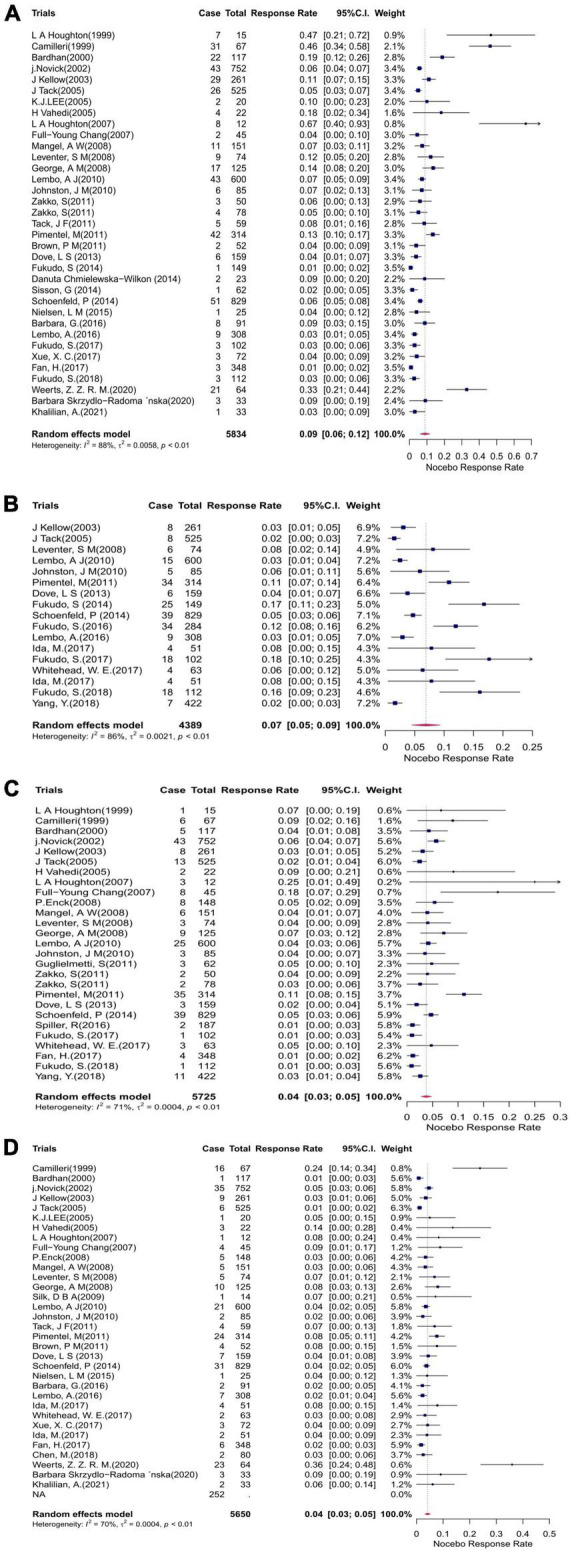
Forest plots showing the cumulative event rates of **(A)** headache, **(B)** nasopharyngitis, **(C)** abdominal pain, and **(D)** nausea in the placebo arms of the clinical trials. CI, confidence interval.

Headache was the most commonly evaluated AE, with 35 studies comprising 432 patients reporting on it. The mean event rate of headache within the studies was 9% (95% CI: 6–12%; *p* < 0.01) (Figure 4A). Nasopharyngitis was the second most commonly reported AE, with 17 studies comprising 244 patients reporting on it. The mean event rate of abdominal pain was 7% (95% CI: 5–9%; *p* < 0.01) (Figure 4B). Abdominal pain was also among the most commonly reported AEs, with an event rate of 4% (95% CI: 3–5%; *p* < 0.01) in 27 studies comprising 249 patients (Figure 4C). In 33 studies comprising 252 patients, the mean event rate of nausea was 4% (95% CI: 3–5%; *p* < 0.01) (Figure 4D). Relatively lower rates were reported for the remaining outcomes: 3, 3, 2, 2, and 1% for infection, diarrhea, dyspepsia, flatulence, and nervous system disorder, respectively.

### Subgroup analysis

Subgroup analysis was conducted according to different study characteristics extracted from the included trials. In terms of drugs, nocebo response rate in the probiotics (16%, 95% CI 0–26%), antispasmodics drugs (8%, 95% CI 0–13%) and traditional Chinese medicine treatment studies (10%, 95% CI –2–23%) was significantly (*p* = 0.01) lower than that in antibiotics trails (51%, 95% CI 43–59%). In the field of AE collection methods, nocebo response rate in patients recording in diaries (22%, 95% CI 10–33%) was lower than the average, and it was higher in patients record AEs through checkup. Concerning the nocebo response, the duration of trial, the dose of medication, trial location, whether the trial received funding, whether the trial was a multicenter study, and the types of diagnosis were not significantly related to the nocebo response rate for either outcome (Tables 2, 3).

**TABLE 2:** Effect of trial characteristics on magnitude of the nocebo response.

	No. of trials	No. of patients receiving nocebo	Pooled nocebo response rate (%)	95% CI	*I*^2^ (%)	*P*-value for *I*^2^
All trials	36	6,462	32.00	26.00–38.00	99	<0.01
**Roman diagnostic criteria**						
I	2	800	36.00	0.00–85.00	99	<0.01
II	13	2,047	35.00	0.00–47.00	96	<0.01
III	21	3,615	30.00	0.00–38.00	96	<0.01
**Trial location**						
Asia	12	1,265	29.00	0.00–39.00	96	<0.01
Europe	10	550	26.00	0.00–38.00	96	<0.01
North America	9	3,549	41.00	0.00–55.00	100	<0.01
International	5	1,098	32.00	0.00–100.00	96	<0.01
**Number of centers**						
Single center	12	492	24.00	0.00–36.00	94	<0.01
Multicenter	24	5,970	35.00	0.00–43.00	99	<0.01
**Duration of therapy**						
3–8 week	13	1,673	29.00	0.00–41.00	98	<0.01
>8 week	22	4,739	33.00	0.00–41.00	99	<0.01
**Dose schedule**						
q.d	15	1,445	34.00	28.00–40.00	80	<0.01
b.i.d	11	3,233	33.00	0.00–47.00	99	<0.01
t.i.d	10	1,784	29.00	0.00–44.00	98	<0.01
**Types of medication**						
Antispasmodics	3	100	8.00	0.00–13.00	0	0.46
Antibiotic	2	1,137	51.00	43.00–59.00	83	0.02
Antidiarrheal agent	8	1,894	40.00	0.00–52.00	99	<0.01
Cathartic agent	2	534	33.00	20.00–46.00	84	0.01
Prokinetic agents	5	1676	33.00	0.00–100.00	99	<0.01
Probiotics	5	3,58	16.00	0.00–26.00	88	<0.01
Traditional Chinese medicine	2	428	10.00	–2.00–23.00	95	<0.01
Neuromodulator	5	123	39.00	0.00–100.00	83	<0.01
Others	4	212	44.00	0.00–56.00	62	0.05
**Sponsorship**						
Yes	28	5,322	32.00	0.00–40.00	99	<0.01
No	8	1,140	30.00	0.00–46.00	96	<0.01
**Types of diagnosis**						
IBS-C	5	1,171	38.00	0.00–53.00	97	<0.01
IBS-D	15	3,277	39.00	30.00–48.00	99	<0.01
Others	16	2,014	23.00	0.00–33.00	98	<0.01
**AE collection methods**						
Diary	10	1,051	22.00	10.00–33.00	96	<0.01
Checkup	2	1,429	57.00	53.00–60.00	54	0.14
Follow-up	10	2,025	35.00	21.00–48.00	98	<0.01
Multiple ways	2	766	38.00	0.00–84.00	96	<0.01
Unknown	12	1,191	33.00	25.00–42.00	92	<0.01

**TABLE 3:** Moderators of the nocebo response.

Subgroups	Estimate	Se	*z*-value	*P*-value	ci.lb	ci.ub
**Duration**						
1–2 week->8 week	0.05	0.21	0.22	0.83	–0.36	0.45
3–8 week->8 week	–0.04	0.07	–0.63	0.53	–0.18	0.09
Sponsorship	0.02	0.08	0.28	0.77	–0.14	0.18
**Location**						
Europe–Asia	–0.03	0.09	–0.39	0.70	–0.20	0.13
International-Asia	0.02	0.10	0.23	0.82	–0.18	0.23
North America–Asia	0.12	0.09	1.40	0.16	–0.05	0.29
Centers	–0.11	0.07	–1.58	0.11	–0.25	0.03
**Dosing**						
q.d.–b.i.d.	–0.00	0.08	–0.01	0.99	–0.16	0.16
t.i.d.–b.i.d.	–0.05	0.09	–0.60	0.55	–0.22	0.12
**Medication**						
Antidiarrheal agent-antibiotic	–0.11	0.13	–0.85	0.39	–0.36	0.14
Antispasmodics-antibiotic	–0.41	0.15	–2.75	**0.01**	–0.71	–0.12
Cathartic agent-antibiotic	–0.17	0.16	–1.06	0.29	–0.49	0.15
Neuromodulator-antibiotic	–0.12	0.14	–0.85	0.39	–0.40	0.16
Others-antibiotic	–0.07	0.14	–0.47	0.64	–0.35	0.21
Probiotics-antibiotic	–0.34	0.14	–2.46	**0.01**	–0.61	–0.07
Prokinetic agents-antibiotic	–0.18	0.14	–1.29	0.20	–0.44	0.09
Traditional Chinese medicine-antibiotic	–0.41	0.16	–2.51	**0.01**	–0.72	–0.09
**Diagnosis**						
IBS-D-IBS-C	0.02	0.10	0.17	0.87	–0.17	0.21
Others-IBS-C	–0.14	0.10	–1.48	0.14	–0.33	0.05
**Criteria**						
II–I	–0.01	0.15	–0.06	0.95	–0.30	0.28
III–I	–0.06	0.14	–0.44	0.66	–0.35	0.22
**AE collection methods**						
Diary-checkup	0.01	0.03	0.38	0.70	–0.04	0.06
Follow-up-checkup	0.01	0.03	0.47	0.64	–0.04	0.06
Multiple ways-checkup	0.02	0.03	0.54	0.59	–0.04	0.07
Unknown-checkup	0.01	0.02	0.52	0.60	–0.03	0.05

Bold term in *P*-value column means *P* < 0.05.

### Sensitivity analysis

Sensitivity analysis (see Supplementary Figure 2) showed that the random exclusion of any study did not lead to a significant change in the pooled nocebo response rate, indicating the stability of the results.

### Publication bias

**FIGURE 5:**
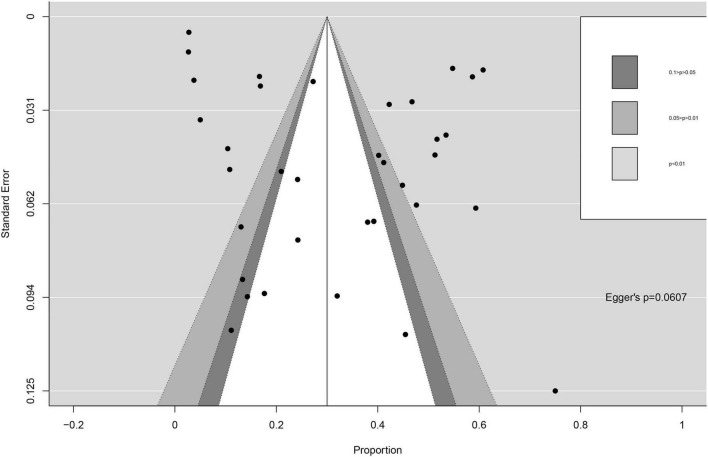
Funnel plots and Egger’s regression test.

The pattern of the funnel plot is symmetrical. Analysis of publication bias by the trim and fill method did not lead to the exclusion of any paper. There is no difference (*p* = 0.0607) in Egger inspection (Figure 5).

## Discussion

We found that serious AEs were 0.74% while the overall incidence of AEs has reached 32%. Several AEs that were more common than other AEs. Besides, nocebo effect response rate changed with different medicine types and different AE collection methods. The year of publication, study region, the duration of the study, the number of daily drug doses, type of IBS, whether the trial was a multicenter study, and whether the trial received funding had no significant effect on the nocebo effect.

In our study, the incidence of AEs and severe AEs in the placebo group was 32 and 0.74%, indicating relatively significant nocebo effects in the RCT of clinical drugs for IBS. This may indicate that RCT has some limitations in evaluating adverse effects of drugs for IBS under the interference of nocebo effect which is caused by the patient’s expectations and has no relations with the physiological effects of the treatment (12). RCTs themselves may lead to the development of negative treatment expectations. AEs may occur due to the nocebo response rather than tested drugs. This result is also consistent with the previous study conclusions drawn by Myers et al. and Christopher et al. in the nocebo effect of RCT trials of clinical drugs for other diseases (13, 14). The phenomenon should not be ignored, and should also be taken into account in the design of subsequent experiments. We believe it will be of great benefit to adopt Real World Research (RWR) in the study of Adverse Drug Reaction (ADR) or use it as a complement.

Of all the AEs reported in our study, headache was the highest (9%), followed by nasopharyngitis (7%), and then abdominal pain and nausea (4%), which is also roughly the same proportion of AEs as Cassandra M et al.’s study of clinical drugs for Crohn’s disease (15). We believe it may be related to the non-specific symptoms experienced by IBS patients. According to some researches, some patients experienced symptoms of IBS after acute intestinal infection, indicating that gastrointestinal infection may be involved in the occurrence of IBS (16, 17). Therefore, it is reasonable to believe the AEs reported in our study, especially gastrointestinal symptoms (abdominal pain and nausea, for example), may be the results of untreated inflammations. For this reason, it is necessary to assess in advance whether patients have symptoms of non-specific AEs before entering trials, so as to avoid misattribution of future symptoms. Researchers can carefully inquire and record whether the patient has a history of headache (including its frequency and degree) and whether the patient has a history of gastrointestinal infection. It is are also encouraged for researchers to evaluate the patient’s nasopharyngeal health status. Patients without the above non-specific symptoms can be preferentially selected to participate in the clinical trials of IBS drugs. Changes in these symptoms should also be recorded after the trial in order to accurately assess the nocebo effect.

Our subgroup analysis showed that the nocebo response rate was higher than average in antibiotic treatment studies, while the nocebo response rate was lower than average in probiotics, antispasmodics, and traditional Chinese medicine treatment studies. As nocebo effect may be the result of previous adverse treatment experience (18), this phenomenon may suggested to be related to the abuse of antibiotics and patients’ unpleasant experience of the adverse effects of antibiotics. Differently, probiotics and Traditional Chinese medicine, which are not classified as the routine treatment of IBS, may bring positive psychological implications and lower expectations of adverse experience to patients (5, 19), resulting in a lower incidence of nocebo effect than the average level. Therefore, doctors and researchers are ought to consider the changed nocebo effects of different medicine types when designing RCTs for IBS to improve the trial design scheme and minimize trial errors. In the evaluation of enrolled patients, the physician may ask the patient in detail about their antibiotic using history and make careful choices about patients who have taken antibiotics with excessive frequency and time or at high doses, or who have pulled through relatively multiple serious AEs. Moreover, the design of clinical trials can be improved by sticking to more rigorous blinding and inform patients about studies without disclosing the types of drugs used.

At the same time, it appeared that nocebo response rate in patients recording in diaries was lower than those recording AEs through checkup. This could indicate that objective checkup collects more than self-documenting in respect of the quantity of AEs in placebo arms. Such difference may be caused by the inability to detect and record AEs based on laboratory abnormalities by patients themselves. The vague definition of AEs told to patients and the lack of initiative to record may also play a role. Compared to this, the objectivity and accuracy of hospital instruments reduces the possibility of under-detection of AEs to a certain extent (20). This result suggests that it is encouraged to collect AEs through self-recording after putting patients through vigorous and standard training to minimize nocebo effects.

As we mentioned before, there are extensive studies on clinical treatment drugs for IBS, but due to the widespread existence of nocebo effect which is undesired and always distort the results of the trial by over-exaggerated lack of treatment efficacy or adverse effects, leading to the early terminated trial and (or) decreased patients compliance (21), the accuracy of clinical conclusions remains to be discussed. Thus, in general, we strongly suggest that researchers improve the recognization of nocebo effect and better design clinical trials with the following protocols so as to ensure more accurate evaluation of treatment effect: (1) Proper adoption of research methods: as we discussed earlier, RCT, with its limitation of adverse effects evaluation, should sometimes make the way for RWR or combined with RWR according to the specific research. (2) Careful selection of enrolled patients: patients with generalized symptoms which commonly occur even in healthy people (22) or who have underwent major serious AEs with the experimented drugs or related diseases before or who tend to have unstable mental status with negative expectations are suggested to be ranked with low priority. (3) Careful choice and phrasing of treatment-related information given to patients: It may make more sense not to inform patients of potential AEs that may not be related to treatment or have little clinical significance (23). (4) Strict implementation of blinding. (5) Appropriate methods of data collection: in this context, self-recording is recommend due to its lower nocebo effect compared to other methods for IBS clinical trials on the premise that patients have received formal training.

Although there have been studies on the nocebo effect of digestive diseases in recent years (12, 24, 25), studies on the nocebo effect of RCT for IBS clinical drugs are still sorely lacking. Our study summarized the nocebo effect and its related factors, and conducted a systematic review and meta-analysis of the incidence of AEs in the placebo groups of IBS related RCTs. We believe it has an important influence on further improving clinical trial design, reducing the placebo effect and increasing patients’ compliance, thus providing the basis for more objective drug safety evaluations. Despite the high heterogeneity among studies, our results were still stable and reliable according to sensitivity analysis.

There are some limitations in our study. First of all, although there was no publication bias, most of the studies only reported the rate of AEs without the specific number of AEs due to the limitations of the results reported in the original studies which we excluded. Secondly, the trial drugs we included did not cover all classes of IBS drugs, and occasionally only one single drug was included without other drugs of the same class. This may partly interfere with the results of our subgroup analysis and probably leave out some drugs with potential higher nocebo effects. Thirdly, a considerable part of the symptoms of adverse reaction events are highly subjective, which were affected by patients’ tolerability to unpleasant experience, so the evaluation may not be completely objective and reliable. Last but not the least, several studies have shown that informed consent has a certain impact on nocebo effect (7, 26), but we did not conduct necessary review and record of informed consent in the included study trials, which may also have a certain impact on the determination of nocebo effect. All the above reasons may be the cause of such heterogeneity.

## Conclusion

In summary, we found that nocebo effect was common in RCTs among IBS patients, and headache, abdominal pain, nasopharyngitis, and nausea were the four most common adverse reactions. Concerning the magnitude of the nocebo response rate, we find there is significant difference among trials using different types of medication. Therefore, when designing and conducting clinical trials, the influence of different drug types on nocebo effect should be taken into account to ensure a more accurate evaluation of treatment effect. Also, different AE collection methods contribute to different nocebo response rate in patients. Based on this result, we recommend the researchers pay attention to the methods of recording AEs and carefully analyze the relation to the intervention.

## Data availability statement

The original contributions presented in this study are included in the article/Supplementary material, further inquiries can be directed to the corresponding author.

## Author contributions

RL: methodology, data curation, formal analysis, and writing—original draft. FC: data curation and formal analysis. XuH: data curation and writing—original draft. YF, QP, and XL: data curation. DW: writing—original draft. JL, XiH, and TB: conceptualization, project administration, and writing—review and editing. All authors contributed to the article and approved the submitted version.

## Footnotes

1
https://www.r-project.org/
