# Supplementary figures and images for: Nocebo response intensity and influencing factors in the randomized clinical trials of irritable bowel syndrome: A systematic review and meta-analysis
Source: Front Med (Lausanne). 2022 Dec 20;9:1018713. doi: 10.3389/fmed.2022.1018713 (PMC9807875; doi:10.3389/fmed.2022.1018713)

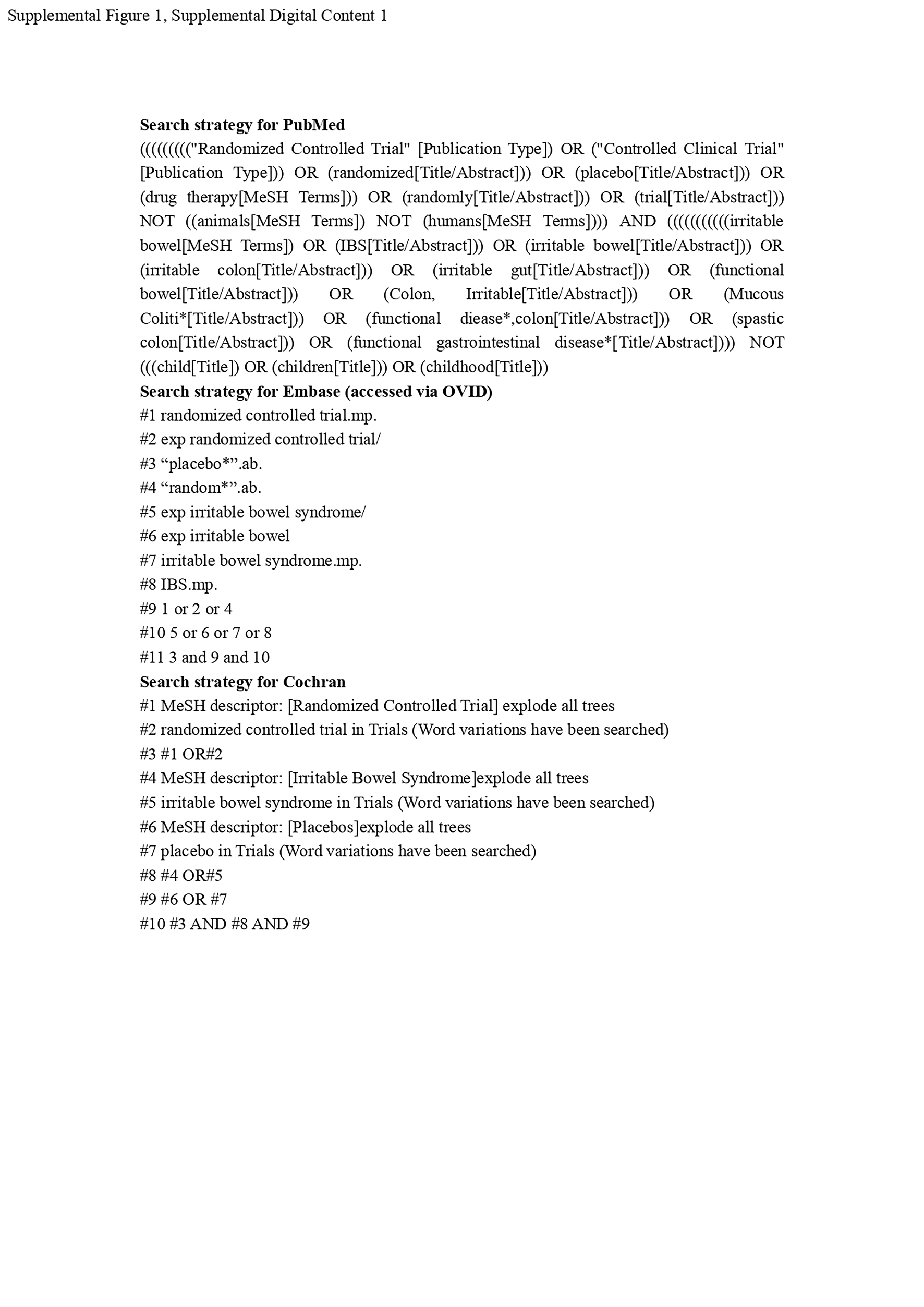

Supplement: Supplementary file 1 [file Image_1.JPEG]

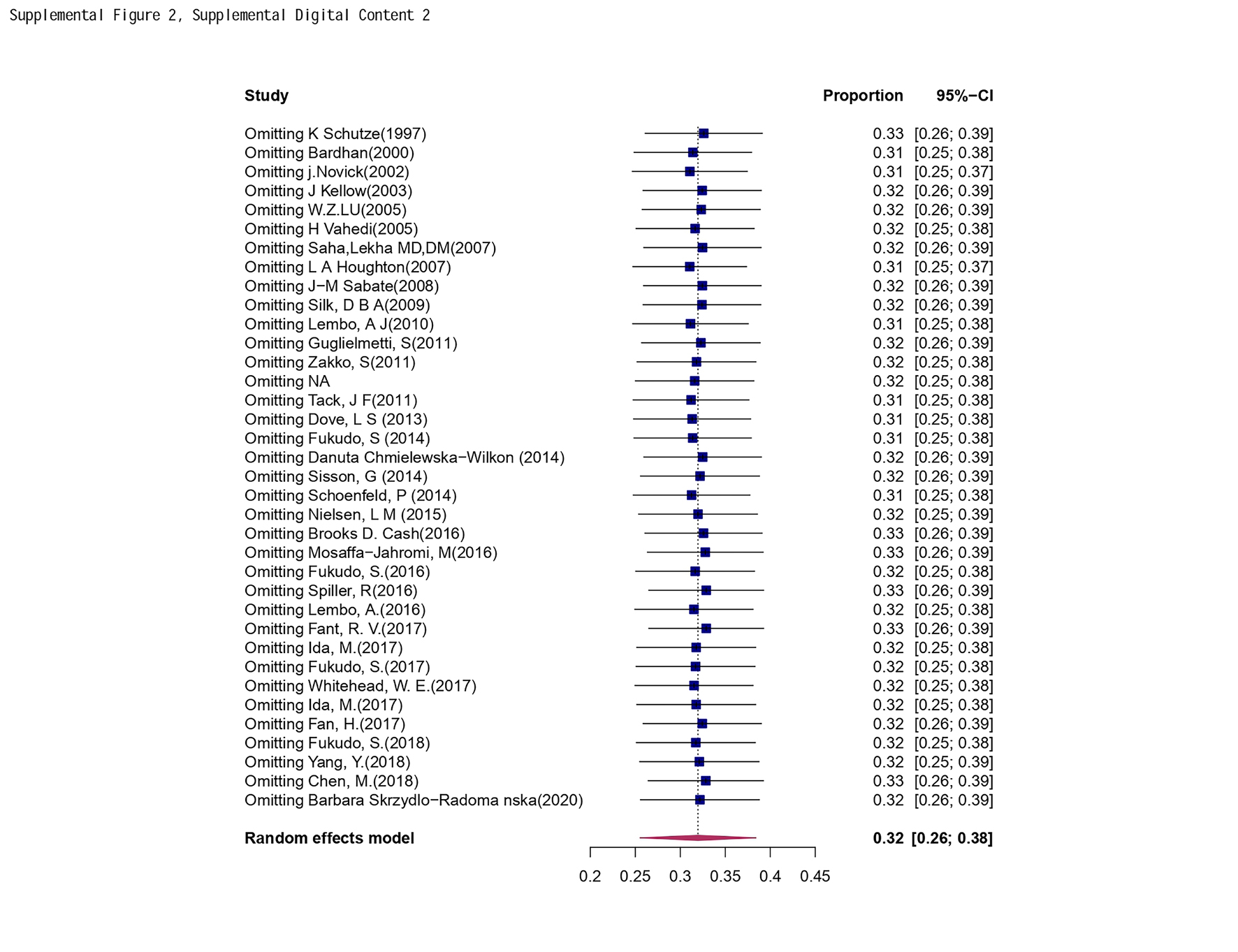

Supplement: Supplementary file 2 [file Image_2.JPEG]
